# Mesenteric Castleman’s disease mimicking neuroendocrine tumour

**DOI:** 10.1016/j.ijscr.2019.09.002

**Published:** 2019-09-18

**Authors:** Ricky H. Bhogal, Andrew Wotherspoon, Aamir K. Khan

**Affiliations:** a Department of Academic Surgery, The Royal Marsden Hospital, Fulham Road, London, SW3 6JJ, United Kingdom; b Department of Histopathology, The Royal Marsden Hospital, Fulham Road, London, SW3 6JJ, United Kingdom

**Keywords:** Castleman’s disease, Neuroendocrine tumour, Lymphadenopathy, Small bowel resection

## Abstract

**Introduction:**

Castleman’s disease is a rare entity and is a benign hyperplastic enlargement of lymph nodes. It can occur anywhere within the body but only approximately 50 cases of mesenteric Castleman’s disease have been reported within the literature.

**Case presentation:**

We report a female patient who was thought to have developed a neuroendocrine tumour within the small bowel and a large nodal mass within the mesentery based on nuclear scintigraphy imaging. Following surgical resection the nodal mass found confirmed to be Castleman’s disease.

**Discussion:**

Although rare complete resection of mesenteric Castleman’s disease offer excellent long term outcome although the diagnosis may only be made after surgery.

**Conclusion:**

The diagnosis of mesenteric Castleman’s disease is often made post-operatively but complete surgical resection offers excellent long-term survival.

## Highlights

• Mesenteric Castleman’s disease is a rare pathology.
• It should be included in the differential for abdominal lymphadenopathy.
• Patients have an excellent prognosis after resection.

## 1 Introduction

Castleman’s disease (CD) is a rare and benign lymphoproliferative disorder that can involve single (unicentric) or multiple lymph nodes (multicentric) anywhere in the body. CD can classified into three distinct histopathological types; namely hyaline-vascular type, plasma cell type and mixed type comprising the former two types [1,2]. Unicentric UCD can affect any node within the body but the vast majority of reported cases involve the mediastinum [3]. Mesenteric UCD is very rare presentation and is often difficult to differentiate from other diseases such a tumour [4]. We present a case of mesenteric UCD that on investigations mimicked a neuroendocrine tumour (NET). We present a review of the current literature and the suggested management of mesenteric CD.

## 2 Case history

**Fig. 1:**
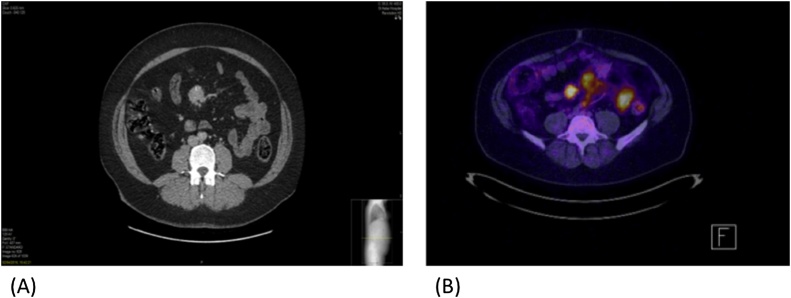
(A) Axial CT demonstrating a nodal mass within the mesentery anterior to the superior mesenteric vessels. (B) NM68 DOTATATE PET scan showing that avidity of the nodal mass and radiological appearances that are in keeping with a neuroendocrine tumour.

**Fig. 2:**
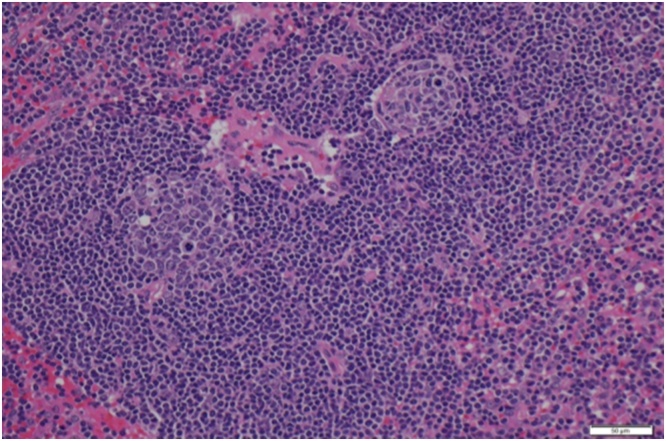
The Mesenteric mass shows lymphoid follicular structures including some with multiple germinal centres. The mantles are traversed by hyalinised vessels. The interfollicular area has reduced cellularity with prominent vessels.

The case is reported as per SCARE recommendations [4]. We report a 43-year old female patient who presented with a 2-year history of gastro-oesophageal reflux that had become more severe recently. She had no previous medical history and was on no regular medication. An upper gastrointestinal endoscopy demonstrated Heliobacter pylori negative gastritis only. Due to the persistence of symptoms she underwent cross sectional imaging in the form of Computed Tomography (CT) that demonstrated a 3 cm nodal mass overlying the superior mesenteric vessels (Fig. 1A). These features were thought to be consistent with a NET and therefore the patient underwent NM68 DOTOTATE PET. This demonstrated avid disease in the mesenteric lymph nodes corresponding to the nodal mass seen on CT (Fig. 1B) although no visible primary tumour could be seen. There was no evidence of metastatic disease on radiological imaging. Gut hormone profile and urinary 5-HIAA were reported within normal parameters. The patient was counselled and after informed consent agreed to undergo laparotomy, small bowel resection and anastomosis. The patient underwent midline laparotomy followed by Cattell-Brasch manoeuvre. The nodal mass was identified in the proximal small bowel mesentery with no primary tumour present. The small bowel was resected (1.05 m) followed by side-to-side anastomosis. The patient made an uneventful recovery and was discharged 6 days after surgery. Histological analysis of the resected mass demonstrated complete excision of the nodal mass with features consistent with CD of the hyaline vascular subtype. In light of these findings the patient was discharged from further follow-up (Fig. 2).

## 3 Discussion

CD was described for the first time in 1954 by Benjamin Castleman and is a benign hyperplastic enlargement of lymph nodes [5]. It remains a rare entity [6,7] and is classified as unicentric (UCD) or multicentric form, depending on the number of lymph nodes involved. The unicentric form represents the most common form (>90%). It tends to occur in the third and fourth decade of life with a slight female predominance with a median age of 35 years [8,9]. The estimated prevalence of CD ranges between 30,000–100,000 in the United States [10].

The aetiology of CD is unknown but chronic low-grade inflammation, immunodeficiency status and autoimmunity have been proposed as putative mechanisms. There appears to be a critical role for inflammatory mediators such as interleukin 6 as demonstrated in preclinical animal models [11]. Dysregulation and overexpression of IL-6 stimulate hepatocytes within the liver to produce acute phase proteins which increase the levels of the hepcidin hormone. IL-6 also stimulates B-cells and blood vessel proliferation promoting the overexpression of the vascular endothelial growth factor and subsequent neoangiogenesis [12].

Although UCD is a not a malignant condition, different malignancies and other diseases are associated with it [[13], [14], [15]]. CD can be classified into three histopathological patterns: a hyaline-vascular (HV) type, a plasma cell (PC) type and a mixed variant. Usually it is the HV type that represents 80–90% of cases and appears more frequently as unicentric disease as in the reported case whereas the PC type is mostly multicentric disease. In the HV variant, lymph nodes involved in the disease, show increased numbers of lymphoid follicles with an increased number of small hyalinized vessels between and within follicles named “lollipop follicles” results in obliteration of medullary sinuses as in the reported patient. UCD usually is identified without symptoms at diagnosis and can be discovered incidentally on radiological imaging as described below. The patients may present symptoms related to the compression of adjacent organs such as vomiting, postprandial discomfort and abdominal or lumbar pain in abdominal-retroperitoneal disease [16,17]. Therefore, because there are no specific symptoms and clinical presentation can vary greatly and a diagnosis of UCD based only upon clinical features is often elusive.

Unicentric disease frequently affects the abdomen-pelvis in only 10% of patients. The location of the disease in mesentery is rare and usually associated with multicentric form unlike the reported case. In a recent case report and literature review [18], only 53 cases of mesenteric UCD were reported worldwide. Preoperative diagnosis is often not achievable. Radiologically, the findings of mesenteric CD are non-specific and the radiological studies alone without histopathological reports will not give a definite diagnosis [19]. CT scans may demonstrate a defined soft tissue density, and the hyaline vascular type is more contrast enhanced than the plasma cell type [19]. Importantly the proximity of the mass to major vasculature, such as in the reported patient, will deter attempts at pre-operative tissue diagnosis with FNAB or FNAC due to the risk of severe bleeding. In these instances surgery and resection is often the preferred route to establishing diagnosis. The laboratory evaluation of patients with UCD should also include immunodeficiency and virological screen to exclude associated pathology.

The radiological findings for UCD are non-specific it is often confused with other lesions such as GIST. Homogeneity with intense contrast enhancement reflecting hypervascularity of the lesion is a characteristic finding at CT of abdominal UCD. Mesenteric UCD commonly appears at CT as a well-defined single mass of soft tissue without satellite nodules or surrounded by normal lymphadenopathy [20,21]. UCD usually results positive on fluorodeoxyglucose PET but no previous cases have reported positivity with NM68 DOTATATE PET. Thus our reported highlights the potential for mesenteric CD to be interpreted, as neuroendocrine tumours on cross-sectional imaging and both should form part of the differential in abdominal masses that are fluorodeoxyglucose PET and NM68 DOTATATE PET positive.

The standard treatment for UCD regardless of histological type is a complete “en bloc” surgical resection, which is a curative approach in almost all cases [22]. When total resection is not possible partial resection can also offer patients benefit and even in these circumstances recurrence rates are low [23]. In rare instances when diagnosis is established pre-operatively an aggressive/radical resection may not be recommended because of associated morbidity and mortality [24]. UCD can also be treated with radiotherapy/chemoradiotherapy, steroids and/or immunotherapy (interferon α and anti-IL-6 antibodies) as an alternative to surgery or after surgery with good results in the case of radiotherapy [25,26]. Although there are conflicting results in the literature, it is commonly agreed that these modalities are not a definitely curative therapeutic option [24]. In the presence of unresectable UCD, neoadjuvant rituximab and neoadjuvant radiotherapy can allow resection to be performed with a lower rate of morbidity given that these treatments may result in mass shrinkage and reduced vascularity [19]. In conclusion, although a rare disease, UCD should always be considered when a solid asymptomatic abdominal mass is incidentally found whilst pre-operative diagnosis is not possible surgery offers long-term remission.

## 4 Conclusion

The diagnosis of mesenteric Castleman’s disease is often made post-operatively but complete surgical resection offers excellent long-term survival.

## Sources of funding

Not applicable.

## Ethical approval

For this study ethical approval is exempted by my institution (The Royal Marsden NHS Trust) and therefore is not applicable.

## Consent

The patient has consented to publishing case report.

## Author’s contribution

RHB – literature review, writing manuscript and editing.

AW – histological analysis.

AZK – writing the manuscript and critical review.

## Registration of research studies

Not applicable.

## Guarantor

Ricky H. Bhogal.

## Provenance and peer review

Not commissioned, externally peer-reviewed.

## Declaration of Competing Interest

The authors have no conflict of interests to declare.
